# Why We Should Bathe

**Published:** 1888-08

**Authors:** 


### WHY WE SHOULD BATHE.

Among all the appliances for health and comfort to mankind we may safely say there is nothing so well known, so useful, and withal so comforting, and yet so little practiced, so carelessly and thoughtlessly neglected, as judicious bathing. The skin of the human body, from head to foot, is a network of pores, which ought always to be kept free and clear of obstructions. These pores are the openings into minute tubes or channels, which lead through unseen meanderings into the sanctum of life within.

To those blessed with good health, a bath as a common sense appliance, gives thrift and grewth to healthy functions, a brightness and delightful serenity, a clearness of mind and buoyancy of spirit. It is certainly a blessing to both mind and body. For the mental worker, it is a nerve tonic. A thorough immersion in water of proper temperature will calm and give strength and tone to his whole system. The indoor laborer who gets but a scanty supply of fresh air, needs a bath to obtain those invigorating elements so common in the open air.

The outdoor laborer—especially the farmer—who works with heroic energy all day long, unavoidably gathers on the entire surface of his body a complete prison-wall of dust and thickening, gummy perspiration ; and when his day's work is done he needs then, more than any other thing, not only a wash, but a good, luscious, *full bath* to fit him for a clean bed and a refreshing sleep.

The glutinous mass of perspiration, dust and filth, which gathers on the surface of the body naturally covers and clogs the pores and often enters them and poisons the system. To remove that filth, frequent ablutious and occasional immersion in water are exceedingly desirable, and usually indispensable to health and comfort ; consequently, every family should have a convenient bath—and a full bath too—of some kind, not only for general neatness of person, so desirable to every individual of taste and culture, but as a means of preserving health, and in many cases, especially under the advice of a good physician, as the safest, pleasantest and one of the most powerful and efficient means of combating disease. Directed by good judgment and wise counsel, a bath is a valuable auxiliary to other remedies, and it can be used when internal remedies cannot. In the long catalogue of diseases to which flesh is heir, scarcely one can be named in the treatment of which a bath is useless. In an emergency, which often happens when least expected, as in cholera, cholera infantum, cholera morbus, cramps, fits, etc., a pliable, portable bath, which requires but little water, ready just at the right time, may save some precious life.

Finally, everyone needs a bath at times, and every human habitation should contain something for a complete immersion in water, and since convenient and efficient portable baths at comparatively low figures are now extensively advertised for sale, there is little cxcuse for anyone to be without this priceless benefit.—*Common Sense Practioner.*

